# *Salmonella* Biofilms Tolerate Hydrogen Peroxide by a Combination of Extracellular Polymeric Substance Barrier Function and Catalase Enzymes

**DOI:** 10.3389/fcimb.2021.683081

**Published:** 2021-05-19

**Authors:** Mark M. Hahn, Juan F. González, John S. Gunn

**Affiliations:** ^1^ Center for Microbial Pathogenesis, Abigail Wexner Research Institute at Nationwide Children’s Hospital, Columbus, OH, United States; ^2^ Infectious Diseases Institute, The Ohio State University, Columbus, OH, United States; ^3^ Department of Pediatrics, The Ohio State University College of Medicine, Columbus, OH, United States

**Keywords:** *Salmonella*, extracellular polymeric substances (EPSs), biofilms, innate immunity, hydrogen peroxide, chronic infection

## Abstract

The ability of *Salmonella enterica* subspecies *enterica* serovar Typhi (*S.* Typhi) to cause chronic gallbladder infections is dependent on biofilm growth on cholesterol gallstones. Non-typhoidal *Salmonella* (e.g. *S.* Typhimurium) also utilize the biofilm state to persist in the host and the environment. How the pathogen maintains recalcitrance to the host response, and oxidative stress in particular, during chronic infection is poorly understood. Previous experiments demonstrated that *S*. Typhi and *S*. Typhimurium biofilms are tolerant to hydrogen peroxide (H_2_O_2_), but that mutations in the biofilm extracellular polymeric substances (EPSs) O antigen capsule, colanic acid, or Vi antigen reduce tolerance. Here, biofilm-mediated tolerance to oxidative stress was investigated using a combination of EPS and catalase mutants, as catalases are important detoxifiers of H_2_O_2_. Using co-cultured biofilms of wild-type (WT) bacteria with EPS mutants, it was demonstrated that colanic acid in *S*. Typhimurium and Vi antigen in *S*. Typhi have a community function and protect all biofilm-resident bacteria rather than to only protect the individual cells producing the EPSs. However, the H_2_O_2_ tolerance deficiency of a O antigen capsule mutant was unable to be compensated for by co-culture with WT bacteria. For curli fimbriae, both WT and mutant strains are tolerant to H_2_O_2_ though unexpectedly, co-cultured WT/mutant biofilms challenged with H_2_O_2_ resulted in sensitization of both strains, suggesting a more nuanced oxidative resistance alteration in these co-cultures. Three catalase mutant (*katE*, *katG* and a putative catalase) biofilms were also examined, demonstrating significant reductions in biofilm H_2_O_2_ tolerance for the *katE* and *katG* mutants. Biofilm co-culture experiments demonstrated that catalases exhibit a community function. We further hypothesized that biofilms are tolerant to H_2_O_2_ because the physical barrier formed by EPSs slows penetration of H_2_O_2_ into the biofilm to a rate that can be mitigated by intra-biofilm catalases. Compared to WT, EPS-deficient biofilms have a heighted response even to low-dose (2.5 mM) H_2_O_2_ challenge, confirming that resident bacteria of EPS-deficient biofilms are under greater stress and have limited protection from H_2_O_2_. Thus, these data provide an explanation for how *Salmonella* achieves tolerance to H_2_O_2_ by a combination of an EPS-mediated barrier and enzymatic detoxification.

## Introduction

The pathoadaptive properties of *Salmonella enterica* subspecies *enterica* serovar Typhi (*S.* Typhi) allow the etiologic agent of Typhoid fever to exist in its human host in both the planktonic and biofilm growth states (Parry et al., 2002; Crawford et al., 2010b; Gonzalez-Escobedo et al., 2010; Gonzalez-Escobedo and Gunn, 2013). With at least 14.3 million cases of Typhoid fever claiming the lives of 136,000 individuals each year (Stanaway et al., 2019), there is significant need to eradicate this disease. However, this illness remains endemic in part because individuals with latent infections can unknowingly transmit *S.* Typhi to others by fecal-oral contamination (Parry et al., 2002; Crump et al., 2004; Gonzalez-Escobedo et al., 2010; Ruby et al., 2012; Kirk et al., 2015). In fact, chronic carriers represent the only known biological reservoir of *S.* Typhi (Ruby et al., 2012; Stanaway et al., 2019) and there is a clear link between biofilm growth on gallstone surfaces in the gallbladder and chronic carriage (Crawford et al., 2010a; Crawford et al., 2010b; Gonzalez-Escobedo et al., 2010; Gunn et al., 2014; Marshall et al., 2014; Adcox et al., 2016). *Salmonella enterica* subspecies *enterica* serovar Typhimurium (*S.* Typhimurium) also forms environmental biofilms and biofilms on cholesterol gallstones/cholesterol surfaces and causes chronic disease in mice similar to *S.* Typhi chronic infections in humans (Coburn et al., 2007; Crawford et al., 2010b; Moraes et al., 2018; Sakarikou et al., 2020). *S.* Typhimurium and other non-typhoidal serovars also form biofilms at intestinal sites and, in immune-compromised individuals, have the ability to invade and cause systemic disease or establish chronic biofilm infections in distal body sites similar to *S.* Typhi (Barthel et al., 2003; Gordon, 2008; Gordon et al., 2010; Joseph et al., 2016). Thus this non-typhoidal serovar provides a useful model for studying biofilm phenotypes *in vitro* and *in vivo*. Despite an appreciation for how it is transmitted, the mechanism for *S.* Typhi biofilm recalcitrance to the immune response during chronic carriage is poorly understood (Hay and Zhu, 2016).

Throughout its infectious cycle, *S.* Typhi encounters oxidative stress in the host environment. Planktonic *S.* Typhi first encounter products of the oxidative burst upon invasion of M cells at Peyer’s patches and entry into macrophages and neutrophils (Ibarra and Steele‐Mortimer, 2009; Ruby et al., 2012; Behnsen et al., 2015). In a well-documented mechanism, *S.* Typhi is disseminated from the intestine by persistence in the *Salmonella* containing vacuole (SCV) inside macrophages (Hurley et al., 2014; van der Heijden et al., 2015; Kurtz et al., 2017). In response to intracellular *Salmonella* infection, phagocytes generate an oxidative burst through the NADPH oxidase (Imlay, 2003; Hébrard et al., 2009; Rhen, 2019). Superoxide $\left(O_{2}^{-}\right)$ produced by the oxidative burst is weakly reactive and unable to pass through bacterial cell walls; toxicity arises when superoxide dismutates (either enzymatically by superoxide dismutase or spontaneously) to hydrogen peroxide (H_2_O_2_) and molecular oxygen (O_2_) (Farr and Kogoma, 1991; Tsolis et al., 1995; Janssen et al., 2003; Halliwell, 2006; Hébrard et al., 2009; Horst et al., 2010). H_2_O_2_ readily crosses bacterial membranes and enters the cytoplasm where it can undergo Fenton chemistry to form hydroxyl radicals (·OH) that damage macromolecules, such as DNA, proteins, and lipid membranes (Janssen et al., 2003; Hébrard et al., 2009; Horst et al., 2010).

Notably, *Salmonella* have multiple redundancies for mitigating oxidative stress. First and foremost, planktonic *Salmonella* within the SCV use SPI-2-encoded T3SS effector proteins to prevent Phox assembly on the SCV membrane, thereby reducing its exposure to oxidative species (Hensel et al., 1998; Vazquez-Torres et al., 2000; Gallois et al., 2001; Holden, 2002). Despite this activity, some oxidative species are still generated leading to a steady state H_2_O_2_ concentration of approximately 1-4 µM and no more than 2 mM in the SCV (Winterbourn et al., 2006; Slauch, 2011; Ortega et al., 2016), which the bacterium mitigates using two classes of enzymes. Peroxiredoxin-type peroxidases (peroxiredoxins) reduce organic hydroperoxides to alcohols and H_2_O_2_ to water at the expense of cellular reducing agents such as NADH and NADPH (Horst et al., 2010). Although peroxiredoxins are limited by the availability of reductants and thus limited in ability to mitigate the oxidative burst, their function is essential to virulence as mutations in genes encoding these enzymes severely limit the ability of *Salmonella* to degrade H_2_O_2_, survive in macrophages, and infect mice (Hébrard et al., 2009; Horst et al., 2010; Ortega et al., 2016). *Salmonella* also has three unique catalase enzymes (KatG, KatE, and KatN), which remain highly catalytic in non-reducing environments and are thought to have a predominant role when H_2_O_2_ concentrations are in the millimolar range (Seaver and Imlay, 2001). However, the role of catalases in SCV-survival is not straightforward as only one of these enzymes is H_2_O_2_-inducible (*katG*, which is transcriptionally-regulated by OxyR) (Pardo-Esté et al., 2018). Contrarily, expression of *katE* and *katN* are growth phase-dependent and occurs at stationary phase as part of the RpoS regulon (Buchmeier et al., 1995; Ibanez-Ruiz et al., 2000; Robbe‐Saule et al., 2001; Robbe-Saule et al., 2003; Hébrard et al., 2009). Furthermore, the role of catalases in planktonic defense against the oxidative burst is dispensable as mutant *Salmonella* lacking all three catalases do not have a reduced growth rate or survival in macrophages and remain virulent in mice (Buchmeier et al., 1995; Hébrard et al., 2009).

These findings raise the question of why *S.* Typhi, an organism characterized by its host specialization through genomic decay and extensive pseudogene formation (Wain et al., 2002; Dagan et al., 2006; Bäumler and Fang, 2013; Langridge et al., 2015; Ortega et al., 2016), would retain multiple redundancies in anti-oxidant function and, in particular, redundancies in catalase enzymes that are not required for planktonic resistance to the oxidative burst. The aforementioned maximum H_2_O_2_ concentration in the SCV of 2 mM is in agreement with our previously-reported minimum inhibitory concentration (MIC) of H_2_O_2_ against planktonic *Salmonella* spp. of 2.5 mM (Hahn and Gunn, 2020). While intracellular planktonic *S.* Typhi are capable of regulating their environment through modifications to the SCV membrane, *S.* Typhi biofilms develop in extracellular environments, such as the gallbladder lumen, and must have additional mechanisms to tolerate environmental oxidative stress. In particular, bile has been shown to be a potent source for oxidative stress and has pleiotropic effects on *Salmonella* gene regulation, membrane protein synthesis, and efflux systems (Gunn, 2000; Prouty et al., 2004; Begley et al., 2005; Merritt and Donaldson, 2009; Walawalkar et al., 2016). Many of these functions are dependent on upregulation of RpoS-dependent general stress pathways (Hernández et al., 2012). In a recent study (Walawalkar et al., 2016), biofilm SOD and catalase pathways were found to be specifically induced in response to ROS stress from bile and induction was dependent on a the autoinducer-2 quorum sensing pathway. Considering the abundance of stationary-phase cells in biofilms, growth- and stress-dependent regulation of catalase genes, and the ability of OxyR to directly sense H_2_O_2_ and induce numerous stress-response proteins, it is logical to expect an important role of *S.* Typhi catalases in biofilm recalcitrance to the host immune environment.

*S.* Typhi biofilms in the gallbladder consist of self-produced extracellular polymeric substances (EPSs) which anchor the biofilm to cholesterol gallstones (Crawford et al., 2010a; Crawford et al., 2010b; Gonzalez-Escobedo et al., 2010; Gunn et al., 2014) and protect resident bacteria from a variety of assaults including antibiotics and host immunity (Scher et al., 2005; Leid, 2009; Kostakioti et al., 2013; Gunn et al., 2016; González et al., 2018; González et al., 2019; Hahn and Gunn, 2020). Our previous study (Hahn and Gunn, 2020) on the innate immune response to *Salmonella* biofilms demonstrated the O antigen capsule, colanic acid, and the Vi antigen are necessary EPSs for *Salmonella* biofilm tolerance to H_2_O_2_. However, further investigation was need to determine the mechanism by which these EPSs resist H_2_O_2_ and protect biofilm resident bacteria from oxidative killing. By using wild-type (WT), EPS-deficient, and catalase mutant biofilms, we have defined the biofilm response to H_2_O_2_ and demonstrated a specific role of EPSs and catalase enzymes in H_2_O_2_ protection.

## Materials and Methods

### Bacterial Strains, Growth Conditions, and Biofilm Sample Preparation

This study was conducted using the *Salmonella* parental WT strains or derivatives of *S.* Typhimurium ATCC 14028 (JSG210) and *S.* Typhi Ty2 (JSG4383) (**Tables 1**, **2**). Tryptic soy broth (TSB) was used for all planktonic and biofilm cultures. When needed, antibiotics were used at the following concentrations: kanamycin (Kan), 45 µg/mL; ampicillin (Amp), 100 µg/mL. Planktonic bacteria were collected from 16-hour overnight broth cultures. Biofilms were initiated and cultured as previously described (Hahn and Gunn, 2020). Briefly, 96-well polypropylene microtiter plates were coated with 500 µg of cholesterol before inoculation to mimic gallstones. When inoculating mixed-strain biofilms (containing WT and mutant strain mixed together), planktonic bacteria were normalized to OD_490_ = 0.65 then diluted 1:12 into mixed culture so that total bacteria starting in the biofilm was equivalent to single-strain biofilms (diluted 1:6). Biofilms were begun by inoculation of 200 µL/well and cultures were maintained at 30°C on a nutator for 96 hours. Supernatants were replaced with fresh media once every 24 hours. Prior to each experiment, biofilm samples were washed 2× with phosphate-buffered saline (PBS) to remove unattached and planktonic bacteria.

**Table 1:** Wild-type (WT) and extracellular polymeric substance (EPS) mutant strains used for this study.

Strain	Genotype	EPS Deficiency	Antibiotic Resistance	Reference Source
JSG210	WT *S.* Typhimurium	–	–	ATCC14028
JSG4581	WT *S.* Typhimurium	–	Amp	This study
JSG3736	*ΔcsgA*	Curli fimbriae	–	(Adcox et al., 2016)
JSG4608	*ΔcsgA*	Curli fimbriae	Kan	This study
JSG3742	*ΔwcaM*	Colanic acid	–	(Adcox et al., 2016)
JSG4583	*ΔwcaM*	Colanic acid	Kan	This study
JSG3672	*ΔyihO*	O antigen capsule	–	(Adcox et al., 2016)
JSG4582	*ΔyihO*	O antigen capsule	Kan	This study
JSG3838	*ΔbcsE*	Cellulose	–	(Adcox et al., 2016)
JSG4609	*ΔbcsE*	Cellulose	Kan	This study
JSG3790	*ΔcsgAΔwcaM*	Curli fimbriae, Colanic acid	–	(Adcox et al., 2016)
JSG4584	*ΔcsgAΔwcaM*	Curli fimbriae, Colanic acid	Kan	This study
JSG3829	*ΔcsgAΔwcaMΔyihO*	Curli fimbriae, Colanic acid, O antigen capsule	–	(Adcox et al., 2016)
JSG4585	*ΔcsgAΔwcaMΔyihO*	Curli fimbriae, Colanic acid, O antigen capsule	Kan	This study
JSG3841	*ΔcsgAΔwcaMΔyihOΔbcsE*	Curli fimbriae, Colanic acid, O antigen capsule, Cellulose	–	(Adcox et al., 2016)
JSG4586	*ΔcsgAΔwcaMΔyihOΔbcsE*	Curli fimbriae, Colanic acid, O antigen capsule, Cellulose	Kan	This study
JSG4383	WT *S.* Typhi *rpoS^+^*	–	–	(Santander et al., 2007)
JSG4587	WT *S.* Typhi *rpoS^+^*	–	Amp	This study
JSG4695	*S.* Typhi *ΔtviB rpoS^+^*	Vi antigen	–	This study
JSG4696	*S.* Typhi *ΔtviB rpoS* ^+^	Vi antigen	Kan	This study

Kan, Kanamycin; Amp, Ampicillin.

**Table 2:** Catalase mutant strains used in this study.

Strain	Background	Mutation source strain	Catalase Deficiency	Antibiotic Resistance	Reference Source
JSG4588	JSG210	SGD_011/012, well A09	Putative catalase protein (ACY88561.1)	Kan	This study
JSG4590	JSG210	SGD_164/165, well E03	*ΔkatE* (ACY88079.1)	Kan	This study
JSG4592	JSG210	SGD_156/157, well B08	*ΔkatG* (ACY91293.1)	Kan	This study

These catalase mutants have no known deficiencies in extracellular polymeric substances. Kan, Kanamycin.

Biofilm aggregates were used in single- and co-culture phenotypic experiments examining the tolerance of EPS and catalase mutant bacteria. To create aggregates, mature biofilms were mechanically collected by scraping microtiter plate biofilms with pipette tips and normalized by total protein quantification (Bradford method) (Hahn and Gunn, 2020). Biofilms used for reverse-transcription quantitative PCR (RT-qPCR) and Western blot experiments were washed 2× with PBS then exposed to H_2_O_2_ without prior disruption. After 1 or 2 hours of H_2_O_2_ exposure, biofilm samples were washed 2× with PBS then mechanically collected in 100 µL PBS for downstream processing (described below). In order to achieve adequate sample yields, each biological replicate was derived by pooling biofilm samples from 32 wells (of a 96-well plate) containing equal biofilm and H_2_O_2_ conditions (summary data presented herein represent a minimum of 3 biological replicates from independent experiments). All t=0 samples were collected immediately after the first PBS wash and thus were never in contact with H_2_O_2_.

### Mutant Generation

Mutation to Vi antigen (*tviB*) in was constructed in *rpoS* ^+^ Ty2 *S.* Typhi (JSG4383) using λ-Red mutagenesis (Datsenko and Wanner, 2000) with the use of primers JG2934-JG2935 (**Table 3**). Briefly, *S.* Typhi carrying the λ-Red recombinase (JSG4393) was transformed with a Kan resistance cassette with *tviB* homology sequence tags. Subsequently, Kan resistance was removed by transformation with pCP20 carrying the FLP recombinase (Cherepanov and Wackernagel, 1995). The deletion was confirmed by PCR using primers JG2936 and JG2937 (**Table 3**) and analysis by gel electrophoresis before temperature-mediated removal of pCP20. Catalase activity in JSG4695 was confirmed to be phenotypically equivalent to the WT (JSG4383) by placing one colony of each strain on a glass slide and exposing to ~20 µL of 3% H_2_O_2_ then observing for reactive bubbling.

**Table 3:** Oligonucleotide primers used in this study.

Primer	Sequence	Purpose
JG2934	**5’—**ATAAAATTTTAGTAAAGGATTAATAAGAGT GTTCGGTATAGTGTAGGCTGGAGCTGCTTC**—3’**	Forward *tviB* sequence tag
JG2935	**5’—**GTCCGTAGTTCTTCGTAAGCCGTCATGATT ACAATCTCACCATATGAATATCCTCCTTAG**—3’**	Reverse *tviB* sequence tag
JG2936	**5’—**TCAGCGACTTCTGTTCTATT CAAGTAAGAAAGGGGTACGG**—3’**	Forward verification *tviB*
JG2937	**5’—**GCTCCTCACTGACGGACGTG CGAACGTCGTCTAGATTATG**—3’**	Reverse verification *tviB*
JG3144	**5’—**AGCAGGAGGCAATATGTT**—3’**	Forward putative catalase protein flanking
JG3145	**5’—**GTCGGAACTCACTTGTCTT**—3’**	Reverse putative catalase protein flanking
JG3147	**5’—**CTGTTTATGCAGGAATCG**—3’**	Forward *katE* flanking
JG3148	**5’—**ATGTCGCATAATGAGAAAT**—3’**	Reverse *katE* flanking
JG3149	**5’—**GGGAGCTGAGATATGAGC**—3’**	Forward *katG* flanking
JG3150	**5’—**AATTAACCTGTCAGATTATTGC**—3’**	Reverse *katG* flanking
JG2081	**5’—**ACGGTCGCGTATGTCCTATC**—3’**	Forward *rpoB* (qPCR)
JG2082	**5’—**GAGTTCGCCTGAGCGATAAC**—3’**	Reverse *rpoB* (qPCR)
JG3165	**5’—**CCGCGAGGTAGCTGGAATAG**—3’**	Forward putative catalase protein (qPCR)
JG3166	**5’—**GTGGGGTCCGATTTCGTTCT**—3’**	Reverse putative catalase protein (qPCR)
JG3167	**5’—**AGCAGAATAGCGACCACTCG**—3’**	Forward *katE* (qPCR)
JG3168	**5’—**CACCCATGAGCAAACGCAAA**—3’**	Reverse *katE* (qPCR)
JG3169	**5’—**TGGTTCCAACTCCGTACTGC**—3’**	Forward *katG* (qPCR)
JG3170	**5’—**TTGCAGATCGAAACGGTCCA**—3’**	Reverse *katG* (qPCR)

### Transduction of Catalase Mutations

The following reagents were obtained through BEI Resources, NIAID, NIH: *Salmonella enterica* subspecies *enterica*, strain 14028s (Serovar Typhimurium) Single-Gene Deletion Mutant Library, Plate SGD_011/012_Kan, NR-29404; Plate SGD_164/165_Kan, NR-42853; Plate SGD_156/157_Kan, NR-42849 (Porwollik et al., 2014). The three catalase mutations (a putative catalase protein, *ΔkatE*, and *ΔkatG*, respectively) were transduced into *S.* Typhimurium (JSG210) by P22 HT-*int* phage transduction. In short, 3 mL of overnight broth cultures of the catalase mutant donors grown in the presence of P22 phage were harvested by the addition of 500 µL chloroform and pelleted at 5000*xg* (5 minutes). Dilutions of the aqueous layer (containing phage lysate) were then used to infect overnight broth cultures of *S.* Typhimurium. Infections were incubated at 37°C for 25 minutes before the addition of LB + 10 mM EGTA and additional incubation at 37°C for 60 minutes. Cultures were spread on LB agar containing 10 mM EGTA and Kan and incubated at 37°C overnight to select for transductants. After two rounds of isolation streaking on selection plates (LB, 10 mM EGTA, Kan) transductants were screened for phage loss on Evans Blue-Uranine plates. Appropriate colonies were selected for genomic DNA isolation (GenElute Bacterial Genomic DNA; Sigma-Aldrich; St. Louis, MO) and confirmed to carry the transduced catalase mutation by PCR amplification using the gene-specific primers JG3144-JG3150 (**Table 3**).

### Antibiotic Markers to Test Mixed-Community Biofilms

WT and EPS mutant strains were differentially antibiotic resistance-marked for use in co-culture experiments. The empty vector plasmids pWSK29 and pWSK129 (carrying Amp^R^ or Kan^R^ cassettes, respectively) were isolated from overnight broth cultures of *E. coli* DH5α (JSG047 and JSG133, respectively) using the QIAprep Spin Miniprep kit (Qiagen; Germantown, MD). The WT of both serovars was transformed with pWSK29 and all mutants were transformed with pWSK129 and selected on LB supplemented with appropriate antibiotics and incubated at 37°C. One resistant colony from each transformation was selected for further use in co-culture experiments (**Table 1**).

### Growth Rate, MIC, and Planktonic Sensitivity to H_2_O_2_

All mutants generated by conjugal transfer, transduction, or transformation were evaluated for growth rate and MIC of H_2_O_2_. Growth rate was determined by 16-hour growth curve conducted in microtiter plates at 37°C from a starting culture of approximately 2.0 × 10^6^ colony forming units per milliliter (CFUs/mL). Growth was monitored by OD_600_ readings every 30 minutes using a SpectraMax M3 plate reader. The MIC of H_2_O_2_ was tested as previously described (Hahn and Gunn, 2020) with starting cultures of 2.0 × 10^6^ CFUs/mL and H_2_O_2_ concentration ranging from 10 mM to 0.156 mM.

Overnight EPS or catalase mutant planktonic cultures were normalized to 2.0 × 10^6^ CFUs/mL, mixed 1:1 with the corresponding WT strain, and sensitivity to 5 mM H_2_O_2_ was evaluated. Cultures were incubated at 37°C and viable CFUs were enumerated at 2 and 3 hours using appropriate antibiotic plates to discriminate between WT and mutant bacteria.

### Aggregate Tolerance to H_2_O_2_

Tolerance of EPS mutant aggregates marked with antibiotic resistance cassettes was evaluated using single-culture biofilms as previously described (Hahn and Gunn, 2020). This test verified that the newly-created strains have tolerance equivalent to the background strains in which they were generated [tolerance of the background strains in single-culture was previously published (Hahn and Gunn, 2020)]. *S.* Typhimurium biofilm aggregates were challenged with 0 mM, 2.5 mM, or 125 mM H_2_O_2_ and *S.* Typhi biofilm aggregates were challenged with 0 mM, 2.5 mM, or 25 mM H_2_O_2_ based on the previously published tolerances differences present between the two serovars in this model.

Biofilm aggregates of catalase mutant bacteria and of co-cultures containing WT and catalase mutants or WT and EPS mutants were also evaluated for tolerance to H_2_O_2_ using the same challenge methods. Challenges were conducted for 2 hours on an orbital shaker (200 rpm) at 37°C. H_2_O_2_ was supplied at 0 mM, 1.25 mM, 12.5 mM, 31.25 mM, or 62.5 mM for single-strain catalase mutant experiments or at 0 mM, 2.5 mM, 62.5 mM, or 125 mM for WT-catalase mutant co-culture experiments. As for single-strain EPS mutant experiments, WT-EPS mutant co-culture experiments were conducted using 0 mM, 2.5 mM, or 125 mM H_2_O_2_ challenges for *S.* Typhimurium strains and 0 mM, 2.5 mM, or 25 mM H_2_O_2_ challenges for *S.* Typhi strains. The values were selected to represent a 0×, 1×, 10×, 25×, or 50× increase from the previously published WT MIC (2.5 mM) (Hahn and Gunn, 2020). Challenge concentrations for catalase mutant biofilms were adjusted to represent proportional fold-differences as these mutants had a reduced MIC. Because co-culture experiments involved strains with differing MICs, all H_2_O_2_ concentrations are expressed henceforth as millimolar. Single-culture experiments were enumerated by serial dilution plating on LB agar and co-culture samples were plated twice on LB + Amp or LB + Kan to independently enumerate WT and mutant bacteria in the sample (respectively).

### Supernatant Transfer

Single-culture biofilms of WT or EPS mutants were started as described. In experiments involving *S.* Typhimurium *ΔwcaM*, *S.* Typhimurium *ΔyihO*, or *S.* Typhi *ΔtviB*, WT biofilms were designated supernatant-source biofilms and mutant biofilms were designated as receiving biofilms. In trials involving *S.* Typhimurium *ΔcsgA* and *S.* Typhimurium *ΔbcsE*, the opposite designations were made. Each time biofilm supernatant was replaced (once every 24 hours), supernatant from the receiving biofilms was removed and discarded. Spent TSB from supernatant-source biofilms was removed by pipetting, filter sterilized (by PES membranes), mixed in a 1:1 ratio with fresh 2× TSB, then used to replenish media on the receiving biofilms. Supernatant-source biofilms received fresh TSB. The PES membranes used are rated to have low protein-binding activity. Mixing spent supernatant with 2× TSB ensured all biofilms received equal nutrient concentrations each day while allowing receiving biofilms to be exposed to soluble factors and waste produced by supernatant-source biofilms. Receiving biofilms were washed and challenged with H_2_O_2_ as described for single-culture biofilm aggregates.

### RNA Isolation

Collected biofilm samples were pooled in 3.2 mL PBS then pelleted at 4,000 rpm (10 minutes, 4°C) and the supernatant was removed prior to freezing pellets at -80°C. RNA was isolated from the frozen pellets using the hot phenol method. Pellets were resuspended in 475 µL AE buffer (50 mM sodium acetate, 10 mM EDTA, pH 5.2) then added to 475 µL phenol and 40 µL 20% SDS. Tubes were incubated at 65°C for 10 minutes, shaking every minute. Samples were then placed on ice (5 minutes) and centrifuged at 10,000 rpm (15 minutes, 4°C) to pellet debris. Aqueous phases were then transferred to new tubes containing 475 µL chloroform, mixed, and centrifuged at 2,000 rpm (10 minutes, 4°C). Second aqueous phases were transferred to new tubes and RNA was precipitated with 500 µL isopropanol and 50 µL 2M sodium acetate. RNA was pelleted at 12,000 rpm (20 minutes, 4°C), washed with 250 µL 70% cold ethanol then re-pelleted at 12,000 rpm (5 minutes, 4°C) before discarding the ethanol supernatant and air-drying on ice for 15 minutes. Finally, pellets were resuspended in 20 µL nuclease-free water (NFW), analyzed for yield, and treated with DNase I (New England Biolabs; Ipswich, MA) for 10 minutes at 37°C according to manufacturer guidelines (protocol M0303). One µL SUPERase-In RNase Inhibitor (Invitrogen; Carlsbad, CA) was added to each 100 µL reaction to stabilize the samples. After the 10 minute incubation, RNA was re-isolated with isopropanol precipitation/ethanol wash as described above then resuspended in 50 µL NFW.

### cDNA Synthesis and Quantitative PCR

RNA was reverse transcribed into cDNA using the SuperScript III First-Strand Synthesis System (Invitrogen; Carlsbad, CA). Initial priming was conducted with 19.25 ng/µL random primers and 769 µM dNTPs in a 13 µL reaction heated to 65°C for 5 minutes then placed on ice for 1 minute. An additional 7 µL cDNA synthesis master mix (prepared for each reaction as: 4 µL 5X Buffer, 1 µL 0.1M DTT, 1 µL Superase-IN, and 1 µL SuperScript-RT III or NFW) was added to each sample which was then incubated sequentially at 25°C (5 minutes), 50°C (60 minutes) then 70°C (15 minutes). The additional step of adding 1 µL (2 units) of the kit-provided *E. coli* RNase H to samples and incubating at 37°C for 20 minutes was conducted to remove RNA remaining complementary to the cDNA.

Catalase gene quantitative PCR (qPCR) was conducted with PowerUp SYBR Green Master Mix (Applied Biosystems; Foster City, CA) and gene specific primers (**Table 3**; 500 nM each). The reference gene was *rpoB*. All samples were run in triplicate using an Applied Biosystems 7500 Real Time PCR System. Copy numbers were calculated by the Livak method (Livak and Schmittgen, 2001).

### Western Blot

Biofilm samples were pooled in 3.2 mL PBS and centrifuged at 4,000 rpm (10 minutes, 4°C). The pellet was resuspended in 60 µL PBS and boiled at 95°C for 10 minutes. Protein concentration was measured by the Bradford method then samples were normalized to 30 µg/µL in Laemmli sample buffer and boiled for an additional 15 minutes. Following a brief vortex and centrifugation to collect tube contents, a total of 750 µg protein from each sample was loaded into the wells of a Criterion TGX stain-free 4-15% gel (Bio-Rad; Hercules, CA). The Precision Plus Protein WesternC molecular weight ladder (Bio-Rad; Hercules, CA) was also included in each gel and proteins were electrophoresed at 200 V for 45-60 minutes until the dye front migrated to the bottom of the gel. Proteins were transferred to a methanol (MeOH)-activated polyvinylidene difluoride (PVDF) membrane (0.45 µm) using Trans-Blot Turbo Transfer System (Bio-Rad; Hercules, CA) set to 2.5A and 25V for 7 minutes. Membranes were blocked immediately after transfer in 5% bovine serum albumin (BSA) prepared in Tris-buffered saline + Tween 20 (TBST) for 1 hour at room temperature. Blocked membranes were probed with polyclonal rabbit anti-catalase peroxidase antibody (Agrisera AS08 374; Vännäs, Sweden) diluted 1:3000 in 5% BSA/TBST for 16 hours at 4°C. The following day, secondary goat anti-rabbit IgG-horseradish peroxidase (HRP) conjugate (Bio-Rad STAR124P) diluted 1:2000 in 5% BSA/TBST was applied along with Precision Protein StrepTactin-HRP conjugate (Bio-Rad; Hercules, CA) diluted 1:5000 for 60 minutes at room temperature. Membranes were washed in TBST 3× (5 minutes each wash) after each antibody incubation. Protein-antibody complexes were visualized with Clarity Western Substrate (Bio-Rad; Hercules, CA) and chemiluminescent signals were captured using a C400 gel imager (Azure Biosystems; Dublin, CA). Protein and background signals were quantified using ImageJ software (Schneider et al., 2012). After background values were subtracted, protein signal values were normalized to baseline conditions (WT protein at t=0, 0 mM H_2_O_2_).

## Results

### EPS-Associated Tolerance to H_2_O_2_ Is Partially a Community Behavior

#### Presence of WT EPSs Protects Some, but Not All, EPS Mutants

**Figure 1:**
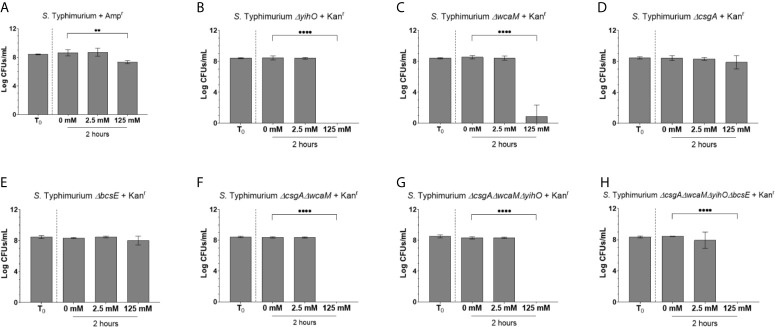
Tolerance of WT *S.* Typhimurium and EPS mutants to H_2_O_2_. **(A–H)** Biofilm aggregates carrying an antibiotic resistance cassette were challenge with H_2_O_2_ at a known tolerable dose (2.5 mM) and a challenge dose (125 mM). Statistical significance was determined by one-way ANOVA with Dunnett correction for multiple comparisons (**p < 0.01; ****p < 0.0001). Each experiment was conducted in triplicate and the data represents the mean of three independent experiments. The error bars indicate SD.

We previously assayed *Salmonella* biofilm tolerance to H_2_O_2_ by testing each WT and EPS mutant against 0 mM, 2.5 mM, 25 mM, 62.5 mM, and 125 mM challenges. In developing this assay, we reported that *S.* Typhimurium EPSs enable biofilm tolerance to H_2_O_2_ at least 50-fold (125 mM) the planktonic MIC (2.5 mM) and that *S.* Typhi EPSs enable biofilm tolerance 10-fold (25 mM). Additional experimentation demonstrated the primary EPSs responsible for this phenotype are the O antigen capsule, colanic acid, and (for *S.* Typhi) the Vi antigen as mutation to these EPSs resulted in loss of tolerance specifically at 125 mM (for EPSs mutated in *S.* Typhimurium) or 25 mM (for EPSs mutated in *S.* Typhi) (Hahn and Gunn, 2020). In order to further investigate these findings, each of the WT and EPS mutant strains were marked with different antibiotic resistances. The introduction of antibiotic resistance had no discernable effect on any of the planktonic phenotypes tested (**Supplementary Figure 1**). The O antigen capsule and colanic acid were again shown to be responsible for biofilm tolerance to H_2_O_2_, which was independent of antibiotic function (**Figures 1A–C**). Furthermore, the elimination of curli fimbriae alone or cellulose alone does not affect tolerance to H_2_O_2_ (**Figures 1D, E**) while multiple EPS mutation eliminated tolerance (**Figures 1F–H**).

While the experiments described above significantly advanced the understanding of the role each EPS has in biofilm tolerance to H_2_O_2_, they did not address the mechanism by which these EPSs protect biofilm-resident bacteria or if the presence of biofilm EPSs may be a community behavior. To this end, antibiotic-marked WT and EPS mutant bacteria were co-cultured in a biofilm. They were then challenged with H_2_O_2_ to determine if the presence of WT EPSs in the biofilm could protect mutant bacteria from otherwise lethal doses of H_2_O_2_ or if the tolerant phenotype is attributed to an individual cell only protecting itself by EPS production. The former result would be indicated by the equal survival of WT and mutant bacteria at 125 mM H_2_O_2_ (normally lethal to the EPS mutant) and suggestive of a community behavior mediated by the production of EPSs, whereas the latter result would be evident by survival of the WT only and indicate the protective EPS must be cell-associated to provide tolerance.

**Figure 2:**
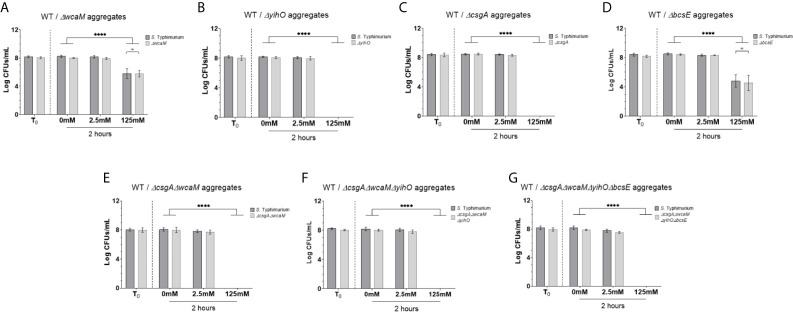
Tolerance of co-cultured WT *S.* Typhimurium and EPS mutant biofilms to H_2_O_2_. **(A–G)** Biofilms were cultured with a 1:1 ratio of WT *S.* Typhimurium and an EPS mutant. Aggregates of these biofilms were challenged with H_2_O_2_ at a known tolerable dose (2.5 mM) and a challenge dose (125 mM) then enumerated on differential antibiotic agar. Significant differences were determined by two-way ANOVA and Tukey method for multiple comparison correction (****p < 0.0001). No significant differences were observed between WT and mutant at any one H_2_O_2_ concentration. Each experiment was conducted in triplicate and the data represents the mean of three independent experiments. The error bars indicate SD.

In order to demonstrate the protective function by WT is specific to the biofilm growth state, planktonic *S*. Typhimurium and *S*. Typhimurium *ΔcsgAΔwcaMΔyihOΔbcsE* were co-cultured in 5 mM H_2_O_2_, which confirmed both strains are rapidly eliminated by a concentration of H_2_O_2_ that is sub-lethal to *S*. Typhimurium biofilms (**Supplementary Figure 1**). To address biofilms, 1:1 mixtures of WT *S.* Typhimurium and EPS mutants were grown and collected as biofilm aggregates for challenge with H_2_O_2._ The equal survival of WT and colanic acid-deficient bacteria (*ΔwcaM*) upon 125 mM H_2_O_2_ challenge indicates production of colanic acid by WT bacteria enhances tolerance to H_2_O_2_ for all biofilm-resident bacteria and that colanic acid does not need to be cell-associated to enact a tolerance phenotype (**Figure 2A**). However, for biofilm aggregates of WT and *ΔyihO* (O antigen capsule), the opposite result was observed in that the WT-produced O antigen capsule was not able to protect the *ΔyihO* mutant from killing upon 125 mM H_2_O_2_ challenge (**Figure 2B**). In fact, even the WT in any aggregate mix with the *ΔyihO* mutation lost its ability to tolerate 125 mM H_2_O_2_ challenge (**Figures 2B, F, G**). Also surprisingly, aggregates of WT co-cultured with curli fimbirae mutants (WT/*ΔcsgA*) or a curli, colanic acid double mutants (WT/*ΔcsgAΔwcaM*) eliminated biofilm tolerance at 125 mM H_2_O_2_ for both WT and mutant bacteria (**Figures 2C, E**). These results were unexpected for two reasons. First, the *ΔcsgA* mutation alone in single-culture biofilm (**Figure 1D**) did not have a discernable effect on tolerance to H_2_O_2_, so it was expected to survive equally with WT when co-cultured. Secondly, co-culture biofilms of WT and *ΔwcaM* (**Figure 2A**) indicate the WT is able to complement the mutant strain with respect to H_2_O_2_ tolerance, so the additional loss of curli fimbriae (*ΔwcaMΔcsgA*) was not expected to compound any tolerance defect from *ΔwcaM*. As expected because of its lack of involvement in H_2_O_2_ tolerance (**Figure 1E**) (Hahn and Gunn, 2020), biofilms co-cultured with WT and cellulose mutant bacteria (WT/*ΔbcsE*) survived challenge with 125 mM H_2_O_2_ equally (**Figure 2D**).

**Figure 3:**
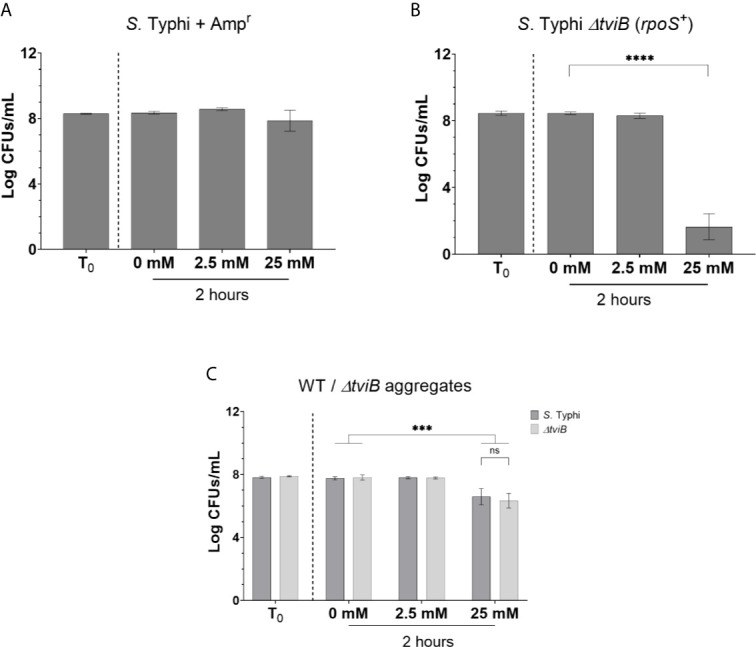
Tolerance of single- and co-cultured *S.* Typhi biofilms to H_2_O_2_. **(A)** WT *S.* Typhi biofilm aggregates carrying an Amp resistance cassette were challenged with H_2_O_2_ at a known tolerable dose (2.5 mM) and a challenge dose (25 mM). **(B)** *S.* Typhi *ΔtviB* biofilm aggregates were challenged in the same conditions as **(A)**. **(A, B)** Statistical significance was tested for/determined by one-way ANOVA with Dunnett correction for multiple comparisons (****p < 0.0001). **(C)** WT *S.* Typhi and *S.* Typhi *ΔtviB* biofilms were cultured in a 1:1 ratio, challenged with H_2_O_2_, and enumerated on differential antibiotic agar. Significance was tested with two-way ANOVA and Tukey correction for multiple comparisons (***p < 0.0005). No significant differences were observed between *S.* Typhi and *S.* Typhi *ΔtviB* at any one H_2_O_2_ concentration. Each experiment was conducted in triplicate and the data represents the mean of three independent experiments. The error bars indicate SD.

Similar to other EPSs, the Vi antigen has an important role in *S.* Typhi biofilm tolerance which is independent of antibiotic resistance (**Figures 3A, B**). Previous investigation of *S.* Typhi biofilms deficient in Vi antigen were conducted using a *tviB* mutant in the Ty2 background that also has a point mutation in *rpoS* (JSG1213), causing RpoS to be non-functional. While many studies have been conducted with *S.* Typhi Ty2, both *Salmonella* catalases and biofilm growth can be affected by RpoS (Fang et al., 1992; Santander et al., 2007; Burda et al., 2018). Thus, we took the additional step to re-construct the *S.* Typhi *ΔtviB* mutation in an *rpoS^+^ S.* Typhi WT (**Table 1**). This change did not alter the tolerance phenotype of *S.* Typhi *ΔtviB* biofilms, which were inhibited by 25 mM H_2_O_2_ (**Figure 3B**). Additionally, introduction of antibiotic resistance genes into each of the *S.* Typhi strains did not cause changes in growth rate (**Supplementary Figure 2**). Equal survival of both the WT and mutant in co-cultured biofilms (WT/*ΔtviB*) challenged with 25 mM H_2_O_2_ indicate WT-produced Vi antigen is sufficient in protecting all biofilm-resident bacteria (**Figure 3C**). As observed previously for *S.* Typhimurium, this result is specific to the biofilm growth state (**Supplementary Figure 2**). Therefore, similar to colanic acid, the presence of Vi antigen in the biofilm serves a community function and protects all biofilm-resident bacteria.

To rule out the possibility that WT biofilm tolerance (and associated compensation in co-culture experiments) is due to non-EPS soluble signaling factors produced by the WT, supernatant transfer experiments were conducted with aggregates from single-culture mutant biofilms receiving filter-sterilized biofilm supernatants. Transfer of WT supernatant to *S.* Typhimurium biofilms deficient in colanic acid (*ΔwcaM*) or O antigen capsule (*ΔyihO*) or on *S.* Typhi biofilms deficient in Vi antigen (*ΔtviB*) had no positive effect on tolerance (**Supplementary Figure 3**). Because the loss of curli fimbriae (*ΔcsgA*) had a negative effect on co-culture tolerance (**Figures 2C, E**), supernatant from the mutant was transferred to the WT before examining WT aggregates for changes in tolerance to H_2_O_2_ (supernatant was transferred from *ΔbcsE* biofilms to WT as a control since cellulose deficiencies were not found to influence aggregate tolerance). Similarly, the negative effect of curli mutations in co-culture biofilms cannot be attributed to a soluble factor released by the mutant acting on the WT (**Supplementary Figure 3**).

#### Bacterial Catalases Have a Role in Biofilm Tolerance

**Figure 4:**
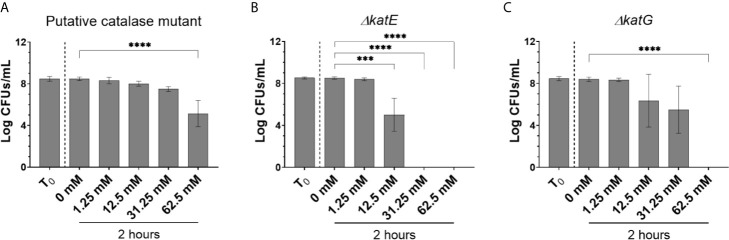
Tolerance of *S.* Typhimurium catalase mutant biofilms to H_2_O_2_. **(A–C)** Biofilm aggregates were challenge with H_2_O_2_ at their planktonic MIC (1.25 mM) and escalating challenge concentrations of 12.5 mM, 31.25 mM, and 62.5 mM H_2_O_2_ to determine the limit of tolerance. Values were selected to represent a 0×, 1×, 10×, 25×, or 50× increase from the experimentally-determined MIC. Significance was determined by one-way ANOVA and Dunnett multiple comparison correction (***p < 0.0005; ****p < 0.0001). Each experiment was conducted in triplicate and the data represents the mean of three independent experiments. The error bars indicate SD.

While there is a clear role for EPSs in tolerance to H_2_O_2_, the contribution for bacterial catalases in detoxifying the biofilm environment was also investigated using three catalase mutant *S.* Typhimurium strains cultured in a biofilm [putative catalase (ACY88561.1), *katE*, and *katG*]. As expected, the H_2_O_2_ MIC was reduced for each of the planktonic catalase mutants while growth rates of each mutant were not altered (**Supplementary Figure 4**). Despite this intrinsic difference, catalase mutant biofilms retained tolerance when challenged with H_2_O_2_, although not necessarily to the same degree as WT biofilm (survival >125 mM H_2_O_2_; **Figure 1A**, **Figure 4**). The putative catalase mutant retained the most tolerance as CFUs were recovered up to 62.5 mM H_2_O_2_ challenge (**Figure 4A**) whereas the *ΔkatE* and *ΔkatG* mutants demonstrated reduced tolerance with no CFUs detectable at 31.25 mM or 62.5 mM H_2_O_2_, respectively (**Figures 4B, C**). Since the catalase mutants have no known EPS differences compared to WT, these data demonstrate that the high degree of tolerance associated with WT biofilms is due to combined action of EPSs and biofilm catalases.

**Figure 5:**
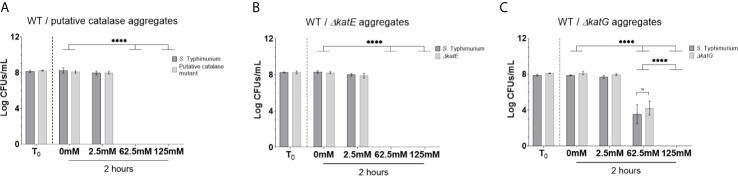
Tolerance of co-cultured WT *S*. Typhimurium and catalase mutant biofilms to H_2_O_2_. **(A–C)** Biofilms were cultured in a 1:1 ratio of WT *S.* Typhimurium and one other catalase mutant. Biofilm aggregates were challenged with H_2_O_2_ at a known tolerable dose (2.5 mM) and two challenge doses (62.5 mM and 125 mM) representing tolerance limits for the catalase mutants and WT *S.* Typhimurium, respectively. Differential antibiotics were used for enumeration and significant differences were identified using two-way ANOVA and Tukey method for multiple comparison correction (****p < 0.0001). No significant differences were observed between WT and mutant at anyone H_2_O_2_ concentration. Each experiment was conducted in triplicate and the data represents the mean of three independent experiments. The error bars indicate SD.

To determine if WT catalase activity could compensate for catalase mutant bacteria in a biofilm, mixed-strain biofilms (WT/catalase mutant) were challenged with 62.5 mM and 125 mM H_2_O_2_. These two challenge concentrations are equal to 50-fold the catalase mutant or WT planktonic MIC, respectively and are concentrations where *ΔkatE* and *ΔkatG* biofilms were unable to survive but WT biofilms demonstrated tolerance. Given that the *ΔkatE* and *ΔkatG* mutant biofilms did not survive 62.5 mM H_2_O_2_ challenge in single-culture, we expected their survival only if WT bacteria in the biofilms could compensate for mutant deficiencies. Co-culturing each catalase mutant with the WT in the planktonic state with 5 mM H_2_O_2_ rapidly eliminated all strains at similar rates (**Supplementary Figure 4**). However, co-culturing the WT with the putative catalase mutant or the *ΔkatE* mutant in a biofilm resulted in a generalized loss of tolerance in both the WT and mutants (both were eliminated by challenge with 62.5 mM or 125 mM H_2_O_2)_) (**Figures 5A, B**). Interestingly, the WT/*ΔkatG* biofilms demonstrated a mixed response. WT bacteria in these biofilms were able to partially compensate for the *katG* mutation and permit tolerance of WT and *ΔkatG* resident bacteria at 62.5 mM (a concentration that previously eliminated *ΔkatG* resident bacteria in single-culture biofilms). However, WT cells were no longer able to withstand 125 mM H_2_O_2_ challenge (**Figure 5C**). Taken together, these data suggest each catalase protein contributes to protection of all biofilm resident bacteria and that tolerance is dependent on a certain threshold of catalase activity that WT cells cannot fully restore when they make up only 50% of the biofilm.

### Mechanism of EPS-Mediated Recalcitrance to H_2_O_2_ Assault

The inability for WT cells to fully complement mutant EPS functions and the role of bacterial catalases in biofilm tolerance led to the hypothesis that *Salmonella* biofilms are tolerant to H_2_O_2_ because EPSs slow penetration of H_2_O_2_ into the intra-biofilm space to a rate that can be mitigated by catalases in that location to keep H_2_O_2_ levels tolerable to resident bacteria. However, without sufficient abundance of certain EPSs, H_2_O_2_ diffuses rapidly and accumulates in the intra-biofilm space thereby having more potent effects against all resident bacteria regardless of EPS-producing abilities. To investigate this hypothesis, the biofilm response to H_2_O_2_ challenge was evaluated in real time by gene and protein expression with the prediction that EPS-deficient biofilms would respond to H_2_O_2_ challenge in less time and/or to a greater degree because the H_2_O_2_ was accessing the intra-biofilm space more rapidly.

#### H_2_O_2_ Challenge Increases Catalase Expression in EPS Mutant Biofilms

To test the hypothesis that EPS mutant biofilms allowed H_2_O_2_ diffusion into the intra-biofilm space faster or to a greater extent, WT *S.* Typhimurium and *S.* Typhimurium *ΔcsgAΔwcaMΔyihOΔbcsE* biofilms were challenged separately with H_2_O_2_ and assayed for the catalase response. Because CFU experiments demonstrated that EPS mutant biofilms (*S.* Typhimurium *ΔcsgAΔwcaMΔyihOΔbcsE*) are eradicated by a 2-hour exposure to 125 mM H_2_O_2_, RNA and protein samples were collected at 1 hour (as well as 2 hours) so that potential differences in the biofilm response to either the tolerable concentration (2.5 mM) or the challenge concentration (125 mM) could still be observed.

**Figure 6:**
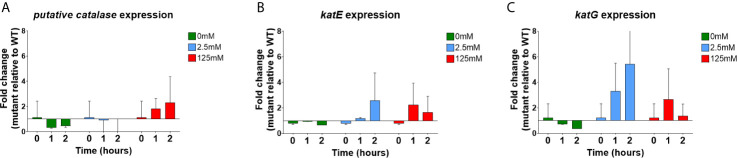
Transcriptional response of catalase genes in WT *S.* Typhimurium and *S.* Typhimurium *ΔcsgAΔwcaMΔyihOΔbcsE* biofilms. qPCR measured copy number of **(A)** putative catalase gene **(B)** *katE* or **(C)** *katG* transcripts present in biofilm cDNA reverse transcribed from total biofilm RNA. RNA samples were collected for WT and EPS mutant at each H_2_O_2_ concentration from 0-2 hours. The data are expressed as fold-change in EPS mutant relative to the WT as determined by the Livak method (Livak and Schmittgen, 2001). Values >1 indicate increased transcription of the target gene by the mutant compared to WT and values <1 represent a decrease. Biofilm RNA was collected and reverse transcribed to cDNA from three independent biofilm challenge experiments. Each cDNA sample was analyzed in triplicate by qPCR to determine C_T_ value of each gene and the data represent the mean of three C_T_ values associated with each independent challenge experiment. Error bars indicate SD.

qPCR was used to assess the transcriptional response of each of the selected catalase genes in the EPS mutant compared to the WT (presented as fold-change of mutant vs. WT; **Figure 6**). For each catalase target, the mutant and WT biofilms had similar levels of expression under control conditions (t=0 and 0 mM) (**Figure 6**). Challenge with 2.5 mM H_2_O_2_ had a minimal effect on the expression of the putative catalase gene (**Figure 6A**). However, this condition led to moderate up-regulation of *katE* in the mutant at 2 hours post challenge and dramatic fold-changes in *katG* transcription at 1 and 2 hours post challenge (**Figures 6B, C**). Challenge with 125 mM H_2_O_2_ led to increased expression of each gene in mutant biofilms though for *katE* and *katG* the overall response was less robust than in 2.5 mM conditions (**Figure 6**). This limited detection was likely due to lethality associated with the 125 mM challenge. Given that KatG is the primary inducible catalase for *Salmonella*, the increased expression observed by 2.5 mM H_2_O_2_ challenge indicates mutant biofilms are experiencing a response consistent with our hypothesis of H_2_O_2_ entering the intra-biofilm space to a much greater extent. Even though the EPS mutant biofilms survive 2.5 mM H_2_O_2_ challenge (**Figures 1** **–** **3**), the induction of *katG* as early as 1 hour and sustained up-regulation at 2 hours indicates a more-stressed population of intra-biofilm *Salmonella* due to limited protection afforded by the EPS-deficient biofilm.

**Figure 7:**
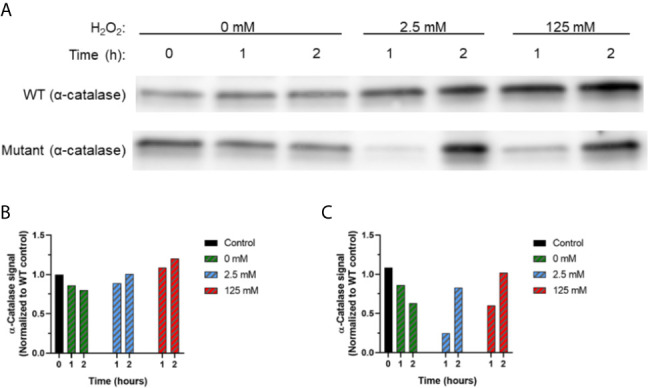
Catalase (translational) response in WT *S.* Typhimurium and *S.* Typhimurium *ΔcsgAΔwcaMΔyihOΔbcsE* biofilms. Western blot was used to analyze total catalase content present in biofilms at each H_2_O_2_ concentration from 0-2 hours. **(A)** Western blot demonstrating general catalase production from WT and EPS mutant biofilms. Densitometry analysis with ImageJ software (Schneider et al., 2012) was used to quantify Western blots of total protein collected from three independent biofilm challenge experiments for **(B)** WT or **(C)** EPS mutant biofilms. Densitometry values were normalized to the WT signal intensity at T_0_, 0 mM.

Western blot assays were conducted to evaluate protein-level differences in biofilm catalases during the same challenge period (**Figure 7**). The limited availability of commercial antibodies only permitted detection of general catalase proteins. Since protein content was normalized prior to loading each gel, gel quantification of each lane was normalized to control conditions (t=0, 0 mM H_2_O_2_). The representative gel (**Figure 7A**) and gel quantification (**Figures 7B, C**) demonstrate that both WT and EPS mutant biofilms had similar catalase content under control (0 mM) conditions from 0-2 hours. While each biofilm responded to H_2_O_2_ exposure by increasing catalase expression, the responses of WT and EPS-deficient biofilms varied greatly. In WT biofilms, a modest increase in biofilm catalase was evident as early as 1 hour after H_2_O_2_ exposure; this response was sustained at 2 hours but only slight increases in catalase were evident for both the 2.5 mM and 125 mM conditions. By contrast, EPS-deficient biofilms had less catalase content after 1-hour exposure to H_2_O_2_ compared to initial conditions. However, exposure to 2.5 mM or 125 mM H_2_O_2_ caused a 3.3-fold or 1.7-fold increase (respectively) in biofilm catalase content from 1 to 2 hours of H_2_O_2_ exposure.

## Discussion

*S.* Typhi produces EPSs to protect biofilm bacteria from many stressors, such as antibiotics and host defenses (Scher et al., 2005; Leid, 2009; Kostakioti et al., 2013; Gunn et al., 2016; González et al., 2018; González et al., 2019; Hahn and Gunn, 2020). We have demonstrated that at least one of these host defenses thwarted by *S.* Typhi biofilms is oxidative stress from H_2_O_2_ and our work furthers understanding of how each of these EPSs protect against this host antimicrobial. Our findings confirmed *S.* Typhi biofilms rely on Vi antigen (**Figures 3A, B**) and *S.* Typhimurium biofilms utilize the O antigen capsule and colanic acid to tolerate H_2_O_2_ doses well-above planktonic-lethal conditions (**Figures 1A–C**). Cellulose, which was demonstrated to be a dispensable EPS (**Figures 1E**, **2D**), must not affect biofilm integrity in the same manner as the other EPSs tested. While EPSs other than Vi antigen may protect *S*. Typhi against H_2_O_2_, this possibility remains to be investigated. Since *S.* Typhimurium biofilms rely on the O antigen capsule for tolerance, we predict this EPS may also contribute to *S.* Typhi tolerance. However, colanic acid will not be a protective EPS in *S.* Typhi, as all *S*. Typhi strains have mutations in colanic acid biosynthetic genes (Nuccio and Bäumler, 2014; Pando, 2017).

To begin investigating the mechanism for EPS-mediated biofilm tolerance, co-culture experiments were used to determine if WT-derived EPSs serve a community function by providing protection for all biofilm resident bacteria regardless of EPS-producing ability. These experiments yielded mixed results in which Vi antigen and colanic acid produced by WT *Salmonella* protected both the WT and mutants deficient in either of their corresponding EPSs (**Figures 2A**, **3C**). These results indicate that WT bacteria produce and secrete/slough off sufficient quantities of Vi antigen or colanic acid for community protection or by sufficient production of other compensatory EPSs by WT and mutants alike. On the other hand, co-culture experiments using WT and O antigen capsule mutants resulted in elimination of both WT and the mutant (**Figure 2B**) indicating the O antigen capsule was not directly protective for the WT cells producing it despite having a critical function in biofilm tolerance. This result suggests that there is insufficient production of the O antigen capsule to protect either the WT or O antigen mutant, or that there is no compensatory EPS production for the lack of O antigen capsule.

The initial finding that *S.* Typhi and *S.* Typhimurium biofilms lose tolerance without Vi antigen or the O antigen capsule (respectively) seemed to be congruent given that both are capsular polysaccharides and that the O antigen capsule has previously been found to have analogous functions in *S.* Typhimurium as the Vi antigen in *S.* Typhi (Sharma and Qadri, 2004; Raffatellu et al., 2005; Gibson et al., 2006; Winter et al., 2008; Marshall and Gunn, 2015; Hiyoshi et al., 2018). However, as discussed, co-culture experiments addressing H_2_O_2_ tolerance demonstrated WT *S.* Typhi was able to compensate for Vi antigen mutants but WT *S.* Typhimurium was not able to compensate for O antigen capsule mutants. This difference highlights a key fitness advantage conferred to *S.* Typhi by its ability to produce Vi antigen. Furthermore, our data suggest *S.* Typhimurium biofilms rely primarily on colanic acid to confer biofilm H_2_O_2_ tolerance as it was the only other EPS included in our analysis that could be fully complemented by the presence of WT in the biofilm. This finding is significant because colanic acid, which is not produced by typhoidal serovars (as mentioned above), is immunostimulatory (Hahn and Gunn, 2020) and its production *in vivo* would defeat the stealth-like behavior of *S*. Typhi. Reliance on colanic acid and not capsular polysaccharide represents a key difference between the two serovars studied here and may suggest why *S.* Typhimurium and other non-typhoidal serovars are less likely to form chronic biofilm infections in human hosts.

Results involving curli fimbriae mutants further complicated our understanding of EPS-mediated protection, as the presence of a *ΔcsgA* mutant in co-cultured WT/*ΔcsgA* biofilms resulted in sensitization of both the WT and mutant despite the fact that biofilms consisting of purely *ΔcsgA* bacteria (and of course the WT) tolerated H_2_O_2_ challenge conditions (**Figures 1D**, **2C**). For curli fimbriae, our rationale that EPSs provide either a benefit only to the bacteria producing the EPS or a community benefit to the whole biofilm does not explain why WT/*ΔcsgA* biofilms have a tolerance defect and represents an active area of investigation. Because curli fimbriae is a major component of biofilm biomass (Adcox et al., 2016), we hypothesize that the co-culture leads to a global change in biofilm properties or H_2_O_2_ susceptibility that are yet to be determined. Similarly, in strains with combinations of EPS mutations (**Figures 2E–G**), all of which have *ΔcsgAΔwcaM* as part of the mutant repertoire, the co-cultured biofilms behave like WT/*ΔcsgA* and not WT/*ΔwcaM* regarding H_2_O_2_ susceptibility. Thus the WT/*ΔcsgA* phenotype, in which both WT and mutant strains become susceptible to H_2_O_2_ at concentrations where they were previously resistant, is dominant. Overall, given the variability between EPS mutants in single- and co-culture biofilm experiments, it is clear that EPS development and biofilm tolerance is governed by more than one pathway. This work is beginning to unveil what is likely a series of complex interactions which must be addressed in the future to enhance understanding of biofilm development and recalcitrance *in vivo*.

The fact that biofilms missing all major EPSs (*S.* Typhimurium *ΔcsgAΔwcaMΔyihOΔbcsE*) still survive up to 62.5 mM H_2_O_2_ challenge (Hahn and Gunn, 2020) indicated additional mechanisms were important for biofilm tolerance against H_2_O_2_. Given the unexplained redundancies of *S.* Typhi catalases, it was logical to focus on this enzyme class. Catalase mutant biofilms have no known EPS deficiencies so the reduced tolerance to H_2_O_2_ in the catalase mutants indicates each of these enzymes are involved in mitigating oxidative stress. KatE is regulated by RpoS in stationary phase. Therefore, it is expected to be present at peak levels only after biofilms have fully developed and, consistently, biofilms with a *ΔkatE* mutation had the greatest reduction in tolerance indicating a pivotal role for KatE in biofilm survival in the presence of H_2_O_2_. Furthermore, the loss of WT tolerance and lack of compensatory activity in WT/*ΔkatE* co-culture biofilms shows that the total amount of the KatE enzyme in the biofilm is essential for the tolerance phenotype and that other catalases cannot make up for this deficiency. Mutation to KatG, which is inducible by the OxyR-mediated stress response, also reduced biofilm H_2_O_2_ tolerance indicating that resident bacteria in mature WT biofilms are able to rapidly sense and induce a protective catalase response *via* KatG when needed (e.g. if KatE present in the biofilms becomes saturated). In further support of this conclusion, WT bacteria in co-cultured biofilms were only able to compensate for *ΔkatG* mutants (**Figure 5**). However, there is still a limit to this inducible activity as 125 mM H_2_O_2_ challenge eliminated WT bacteria in WT/*ΔkatG* co-cultured biofilms and the inducible KatG response presumably occurs (albeit unsuccessfully) in WT/*ΔkatE* biofilms. Taken together, these data indicate a novel function of catalase enzymes that is essential for biofilm tolerance and provides a plausible explanation for why *S.* Typhi has retained multiple catalase enzymes though they are redundant and dispensable during acute infection and planktonic survival in the host (Buchmeier et al., 1995; Hébrard et al., 2009). Consistent with EPS co-culture experiments, we determined catalase-associated tolerance is also dependent on the enzymatic capacity of the biofilm unit and not necessarily the functionality of individual cells within the biofilm.

Finally, to bring a model for biofilm H_2_O_2_ tolerance into focus, it was important to evaluate the role of EPSs and catalases in a unified experiment. This was conducted through qPCR and Western blot examination of the catalase response in WT and EPS mutant (*S*. Typhimurium *ΔcsgAΔwcaMΔyihOΔbcsE*) biofilms. The most prominent response measured by qPCR was from *katG* which was induced in EPS mutant biofilms by both the low and challenge concentrations of H_2_O_2_ (2.5 mM and 125 mM, respectively). This evidence of H_2_O_2_-mediated stress, even at 2.5 mM, shows that the absence of EPSs allows easier penetration of H_2_O_2_ into the biofilm, supporting our hypothesis. Furthermore, the sustained transcriptional response in the EPS mutant from 1 to 2 hours corresponded with a substantial increase in catalase protein 2 hours post challenge indicating the biofilms induce an enzymatic response to H_2_O_2_. Nonetheless, this response is inadequate at protecting biofilm bacteria as demonstrated by CFU experiments involving *S.* Typhimurium *ΔcsgAΔwcaMΔyihOΔbcsE* mutants (**Figures 1** **,** **2**). While unexpected, the reduced catalase content in the EPS-deficient biofilms at 1-hour exposure to H_2_O_2_ could indicate that the weak biofilm-forming ability of the mutant prevents some biofilm-resident bacteria from progressing to stationary phase. This would prevent RpoS-mediated KatE production, leading to fewer total catalases present at experimental onset (further limiting the fitness of the EPS mutant) and predisposing the mutant to the stress-response observed by *katG* induction upon H_2_O_2_ influx. By contrast, the limited increase in catalase proteins from 1 to 2 hours observed for WT biofilms suggests that WT biofilms do not need to induce a large enzymatic response as they rely first on EPSs to maintain a steady state of H_2_O_2_ influx regardless of external H_2_O_2_ concentration (2.5 mM vs. 125 mM) that can be mitigated with existing catalase enzymes. From the CFU experiments (**Figures 1**, **3**) it is apparent that this response, in combination with appropriate EPSs, is sufficient for robust biofilm tolerance.

## Conclusion

We previously reported *Salmonella* biofilms cultured *in vitro* are tolerant to H_2_O_2_. Our work here moves the field forward as it provides an explanation for how *Salmonella* achieves this function using a combination of the physical barrier arising from certain EPSs and enzymatic mitigation. It has been known for quite some time that *Salmonella* EPSs can vary significantly depending on growth conditions and other environmental signals (Scher et al., 2005), however by attributing the tolerance phenotype to specific EPSs, we are able to predict which EPSs are likely to be essential for biofilm survival *in vivo*. We recognize that our challenge concentration of H_2_O_2_ (125 mM) is likely not encountered *in vivo*, although the true microenvironmental H_2_O_2_ concentration encountered by *Salmonella* in the gallbladder environment is not known. However, the ability of the WT biofilm to adequately sense and respond to H_2_O_2_ even at extreme concentrations compared to the stress response observed from EPS mutant biofilms at planktonic-lethal levels indicates that *Salmonella* is well-suited for the host environment because of its biofilm lifestyle and that additional clearance mechanisms must be employed by the host in order to eliminate chronic infections.

## Data Availability Statement

The original contributions presented in the study are included in the article/**Supplementary Material**. Further inquiries can be directed to the corresponding author.

## Author Contributions

All authors contributed to the article and approved the submitted version. The following list described the contributions of each author: Conceptualization, MH and JSG. Methodology, MH, JFG, and JSG. Investigation, MH. Formal analysis, MH and JSG. Writing (original draft preparation), MH. Writing (review and editing), JFG and JSG. Supervision, JSG. Funding acquisition, JSG.

## Funding

This research was supported by the grants R21AI156328, R21AI153752, and R01AI116917 from the National Institutes of Health to JSG and with additional funds provided to JSG by the Abigail Wexner Research Institute at Nationwide Children’s Hospital.

## Conflict of Interest

The authors declare that the research was conducted in the absence of any commercial or financial relationships that could be construed as a potential conflict of interest.
