# Supplementary material for: Salmonella Biofilms Tolerate Hydrogen Peroxide by a Combination of Extracellular Polymeric Substance Barrier Function and Catalase Enzymes
Source: Front Cell Infect Microbiol. 2021 May 19;11:683081. doi: 10.3389/fcimb.2021.683081 (PMC8171120; doi:10.3389/fcimb.2021.683081)
Supplement: Supplementary file 1 [file Table_1.docx]

Supplementary Material

# Supplementary Figures and Tables

## Supplementary Figures


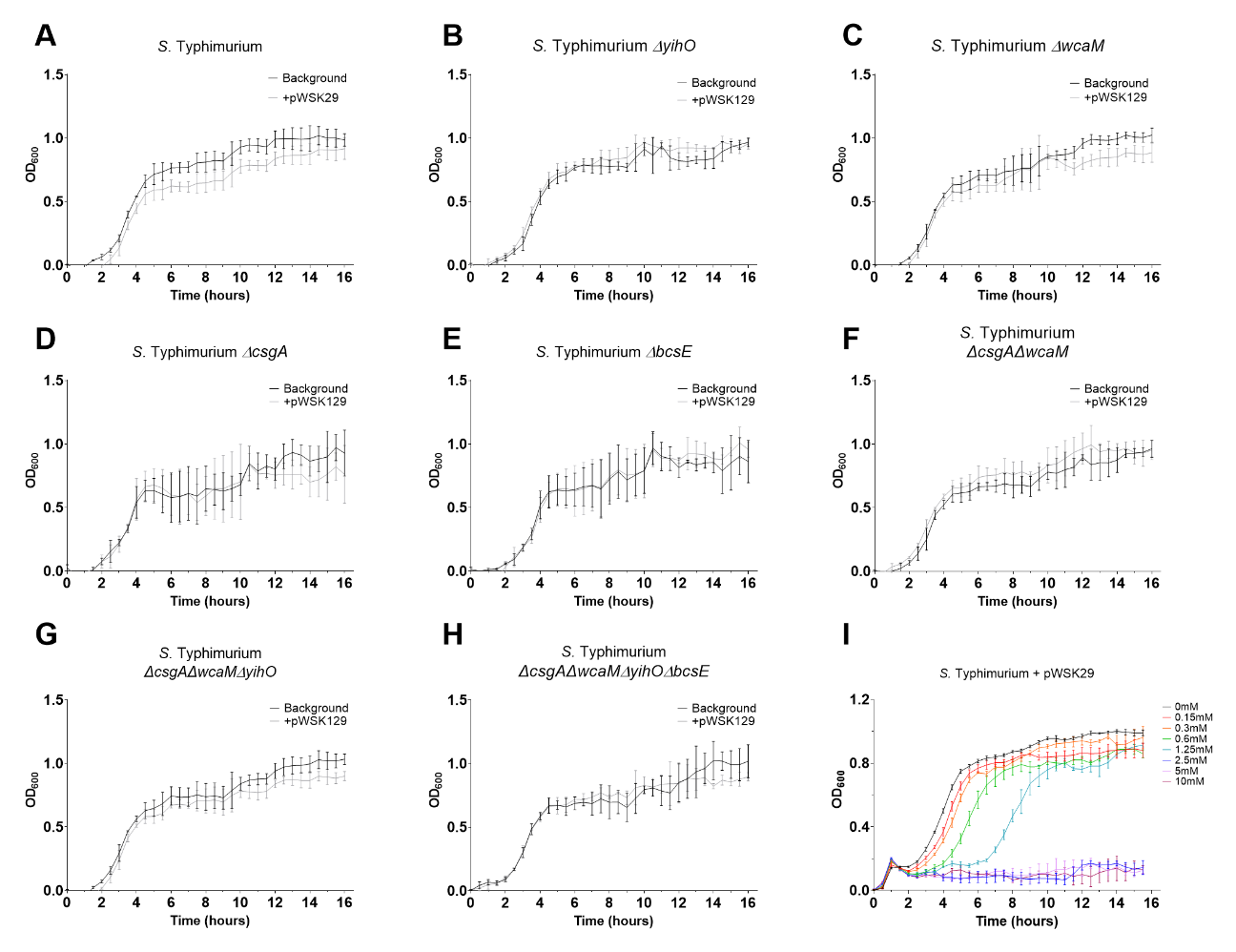

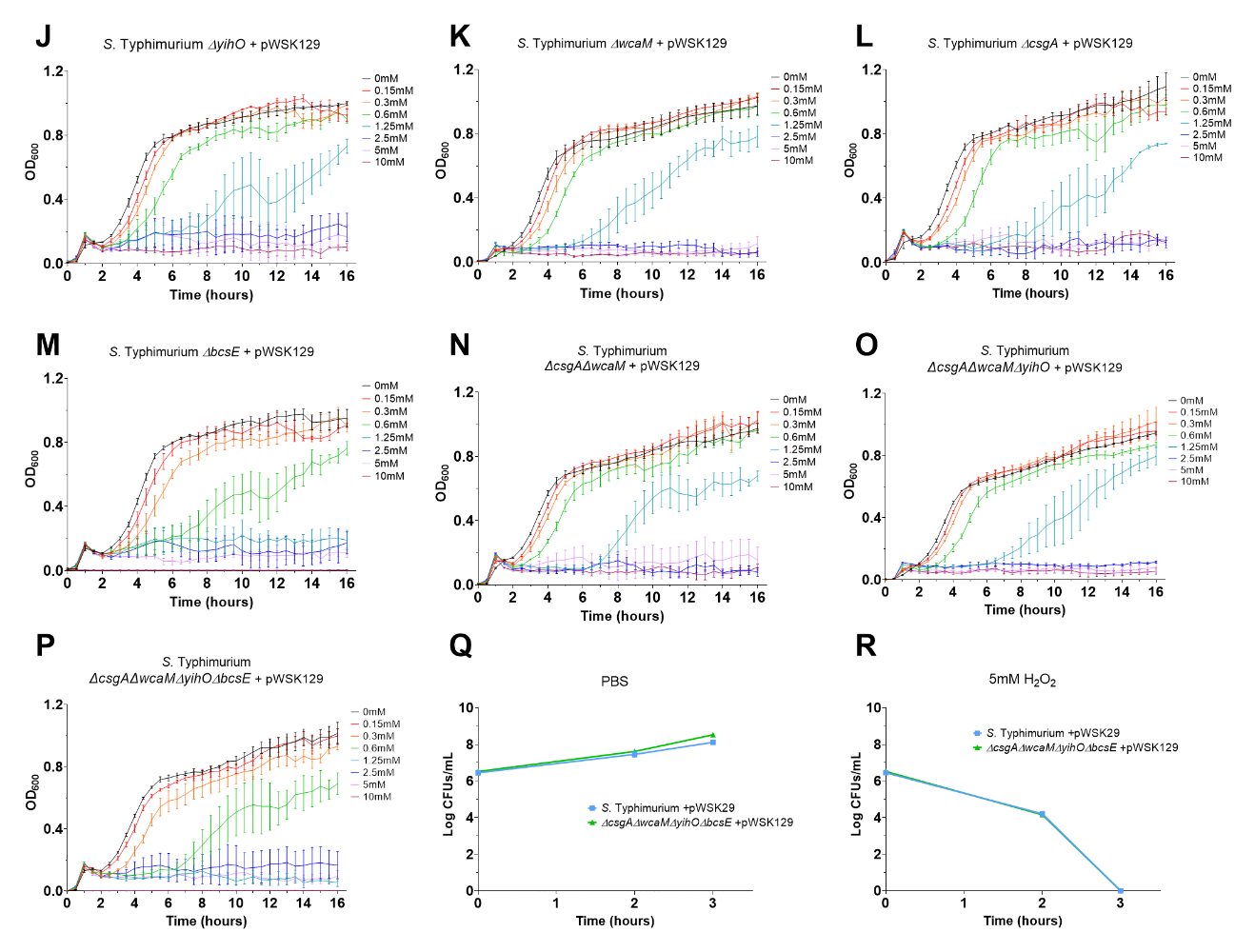


**Supplementary Figure 1.** **Planktonic phenotypes of antibiotic-resistant *S.* Typhimurium strains created for this study.** Previous studies have demonstrated *S.* Typhimurium and EPS mutants have similar planktonic phenotypes in regards to growth rate, and H_2_O_2_ MIC. (**A-H**) Growth rate comparison between newly-created strains (+pWSK29 or +pWSK129) compared to the *S.* Typhimurium strain each plasmid was introduced to (WT or EPS mutants). Using two-way ANOVA and the Sidak method for multiple comparison correction, no significant differences in growth rates were identified. (**I-P**) Antibiotic resistant strains were verified to have the same H_2_O_2_ MIC (2.5 mM) as previously published (Hahn and Gunn, 2020) for non-antibiotic resistant strains. (**Q, R**) Co-culture of planktonic *S.* Typhimurium and *S.* Typhimurium *ΔcsgAΔwcaMΔyihOΔbcsE*. No significant differences in viable CFUs were observed between strains (determined by multiple t-tests). Each experiment was conducted in triplicate and the data is derived from averages of 3 independent experiments. Error bars indicate SD.


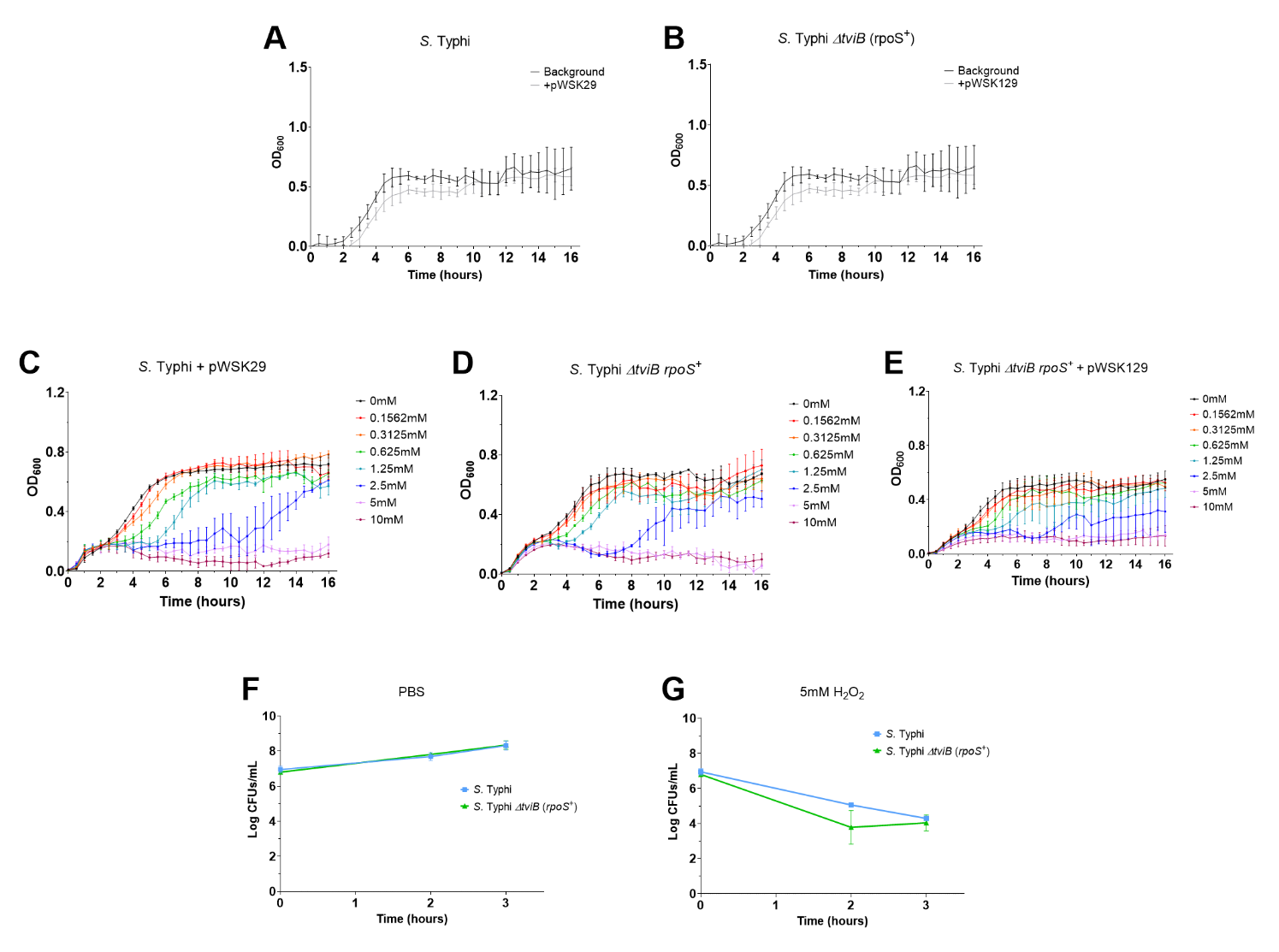


**Supplementary Figure 2. Planktonic phenotypes of antibiotic-resistant *S.* Typhi and *rpoS*-corrected *S.* Typhi *ΔtviB* strains created for this study.** (**A, B**) Growth rate comparison between newly-created strains (+pWSK29 or +pWSK129) compared to the *S.* Typhi strain each plasmid was introduced to (WT or mutant). No significant differences in growth rates were identified by two-way ANOVA with Sidak correction for multiple comparisons. (**C-E**) New *S.* Typhi strains were evaluated for H_2_O_2_ MIC. While growth was observed at 2.5 mM, this shift was not considered consequential because differences in biofilm phenotypes remained apparent at 25 mM H_2_O_2_. (**F, G**) Co-culture of planktonic *S.* Typhi and *S.* Typhi *ΔtviB*. No significant differences in viable CFUs were observed between strains (determined by multiple t-tests). Each experiment was conducted in triplicate and the data is derived from averages of 3 independent experiments. Error bars indicate SD.


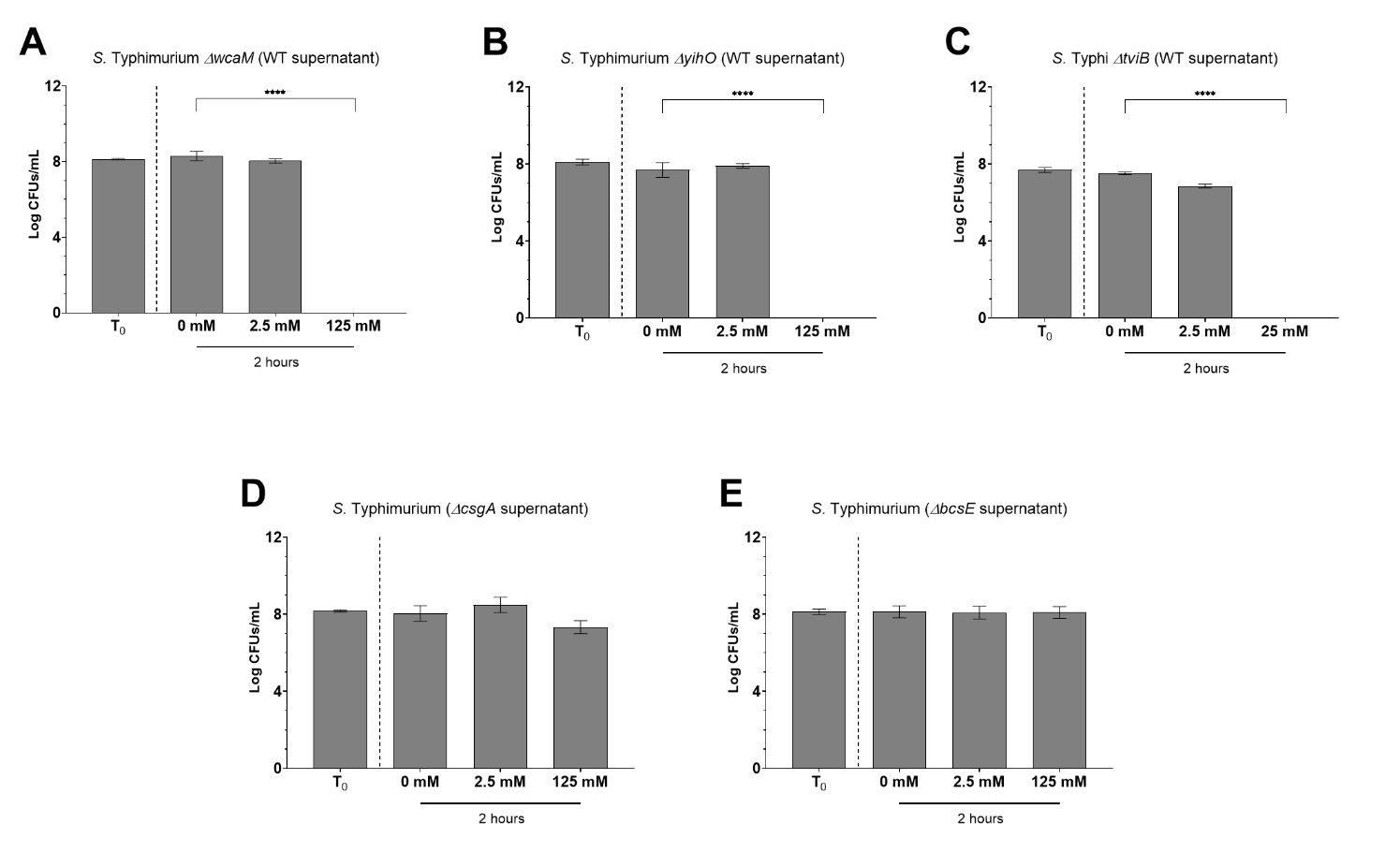


**Supplementary Figure 3. H_2_O_2_ tolerance of supernatant transfer receiving biofilms.** Aggregates from biofilms that received supernatant were challenged with H_2_O_2_ at a known tolerable dose (2.5 mM) and a challenge dose (125 mM). (**A-C**) *S.* Typhimurium *ΔwcaM*, *S.* Typhimurium *ΔyihO*, and *S.* Typhi *ΔtviB* biofilms were cultured with supernatants transfered from respective WT biofilms. (**D, E**) *S.* Typhimurium biofilms were cultured with supernatants transferred from *S.* Typhimurium *ΔcsgA* or *S.* Typhimurium *ΔbcsE*. Statistical significance was determined/tested for by one-way ANOVA with Dunnett correction for multiple comparisons (****, p < 0.0001). Each experiment was conducted in triplicate and the data represents the mean of three independent experiments. The error bars indicate SD.


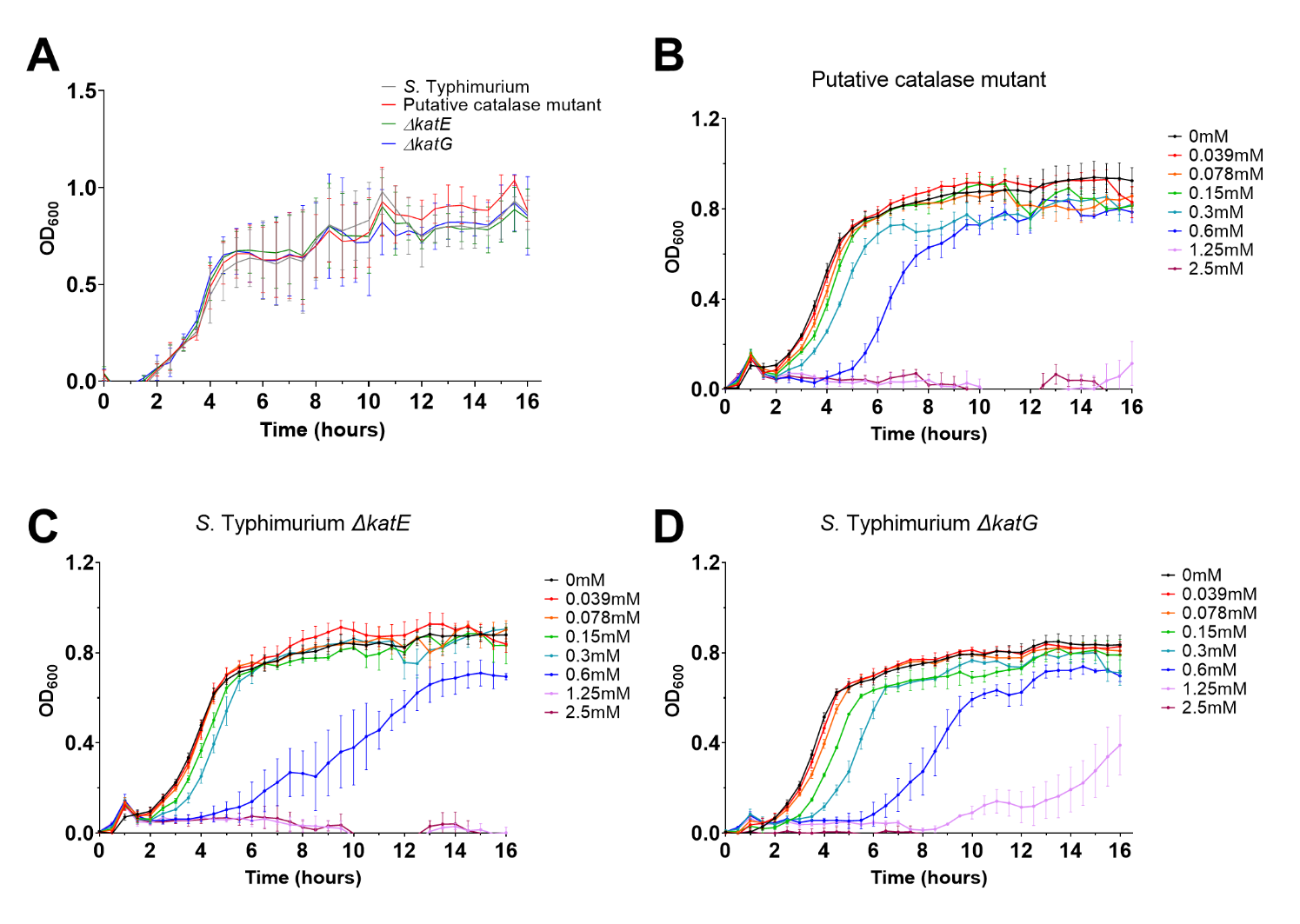

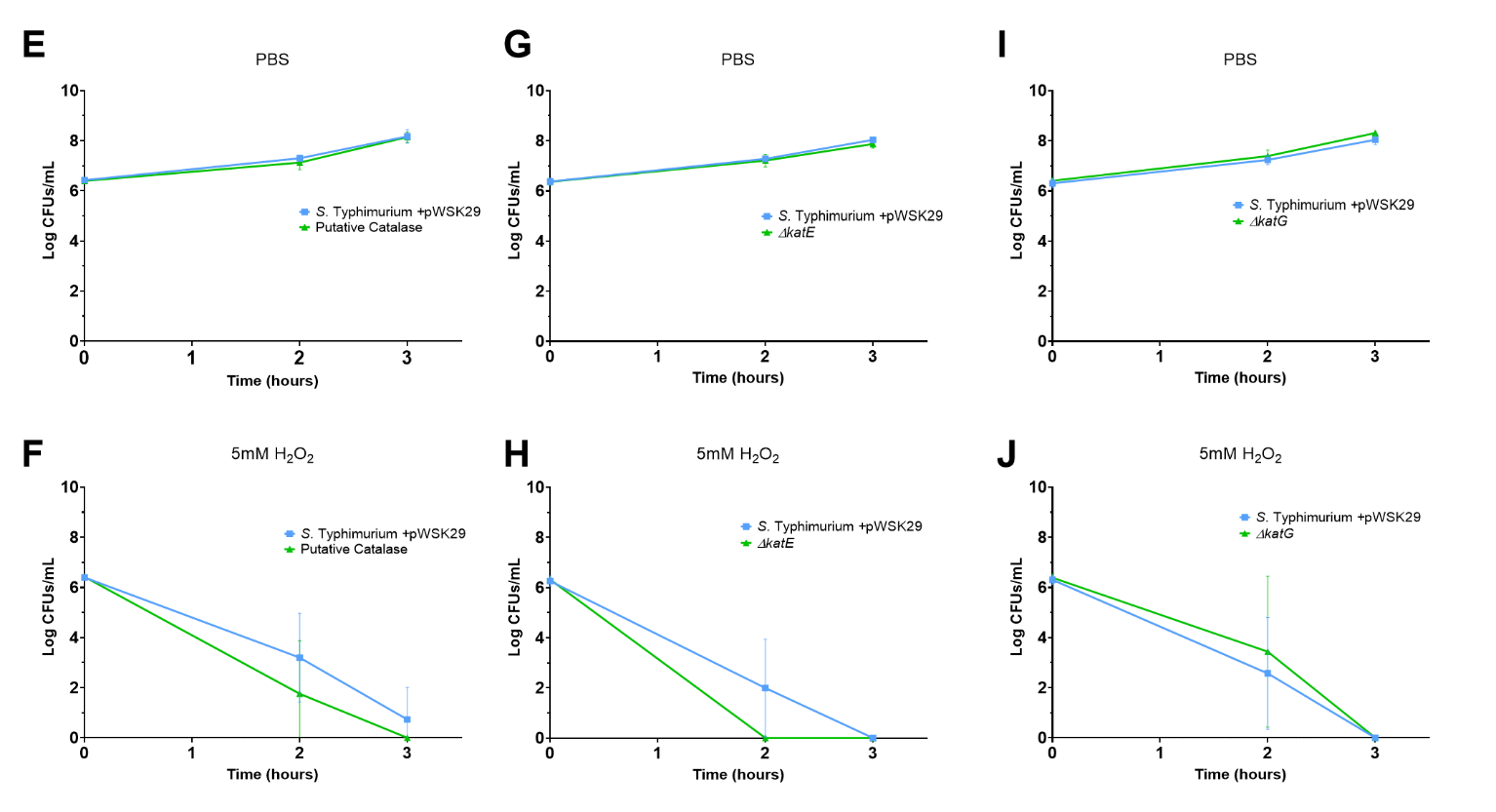


**Supplementary** **Figure 4. Planktonic phenotypes of catalase mutant *S.* Typhimurium strains.** (**A**) Growth rate comparison of each catalase mutant compared to *S.* Typhimurium background the mutations were transduced into. No significant differences in growth rates were identified (determined by two-way ANOVA and the Sidak method for correcting multiple comparisons). (**B-D**) The MIC of H_2_O_2_ was evaluated for each catalase mutant and found to be reduced (compared to WT) from 2.5 mM H_2_O_2_ to 1.25 mM H_2_O_2_ indicating a need to adjust biofilm challenge concentrations when evaluating biofilms containing catalase mutant bacteria. (**E-J**) Co-culture of planktonic *S.* Typhimurium and each catalase mutant. Using multiple t-tests, no significant differences in viable CFUs were observed for WT and each mutant being tested. Each experiment was conducted in triplicate and the data is derived from averages of 3 independent experiments. Error bars indicate SD.
